# Editorial to the *South African Journal of Communication Disorders* special issue

**DOI:** 10.4102/sajcd.v63i2.167

**Published:** 2016-07-28

**Authors:** Heila Jordaan, Ramona Kunene Nicolas

**Affiliations:** 1 Faculty of Humanities, Speech Pathology and Audiology, University of the Witwatersrand, South Africa; 2 Department of Linguistics, University of the Witwatersrand, South Africa

The 2014 annual joint LSSA/SAALA/SAALT Linguistics and Applied Linguistics Conference took place at the University of the Witwatersrand from 24 to 27 June, 2014. The theme of the conference was ‘Synergies and intersections’, with a focus on an interdisciplinary approach to language theory and practice. Featuring over 115 presentations, the conference was primarily held for South African linguists and language practitioners, but it was also well attended by scholars from France, India, New Zealand, Nigeria, Switzerland, the UK and the USA.

Plenary talks ranged from phonological processing, meaning construction in discourse to turbulent diversities in multilingual encounters, multimodal trans-lingual practices and sociocultural processes. Panel sessions focused on reading practices in African languages; the application of corpus linguistics to discourse studies; socio-phonetics in South Africa with a focus on South African, young, black, middle-class English speakers, gesture, language acquisition, culture, and the grammatical investigation of the Afrikaans verb. Postgraduate students and researchers alike enjoyed the interdisciplinary nature of this conference and the enthusiasm around the study of South African languages was infectious.

Against this backdrop, Ramona Kunene-Nicolas from the Wits Linguistics Department and Heila Jordaan from the Wits Speech Pathology and Audiology Department decided to collaborate on one of their research interests: acquisition of South African languages by South Africa–based scholars. Research on the acquisition of South African languages was vibrant in the 1990s with the pioneering works of scholars like Susan Suzman, Katherine Demuth, Mowrer and Burger, and Connelly, to name a few. Thereafter, there was a lull for some 18 years before the contemporary research of Gxilishe, Jordaan, Pascoe and Smouse, Kunene, Bortz and Tshule (all in the 2000s) showed that there was a renewed, robust interest in the much-needed studies of language acquisition within the South African context. Speech-language therapists, who are confronted with a dearth of research in language acquisition for clinical purposes, initiated contemporary research in this domain. Jordaan and Kunene-Nicolas sought to bring together this vibrant and renewed interest by South African scholars in this volume.

It is with this special issue that we hope to advertise the important research that is conducted locally and yet within a global framework. This special issue seeks not only to create awareness of the current work but also to highlight the challenges faced by a multilingual South Africa. This is the beginning of several special issues that will soon tackle bilingualism and multilingualism in order to improve the literacy levels of young South Africans. This special issue also seeks to highlight the need for interdisciplinary work by linguists and language practitioners to find solutions. In this special issue, we showcase some of the most recent research on South African language acquisition.

In South Africa, many children speak different languages at home than their language of instruction. This affects the development of conceptual and analytical skills, expression of thought and reading and writing. A synergy of psycholinguistic and language practice research is crucial in improving pedagogy and clinical diagnosis. Many children with language difficulties or delays often are undiagnosed and this increases their chances of academic failure. Policies devised by politicians and education specialists who have no background in linguistics change their strategies regularly, with a negative impact on teaching and learning.

This special issue seeks to bring together contemporary research on South African languages, with special emphasis on the Bantu language family, Afrikaans and South African English, and it specifically focuses on acquisition using both psycholinguistic and clinical approaches. This pioneering interdisciplinary synergy will allow the exposition of local academic research within the local and the international field of language development.

From a psycholinguistic and linguistic theory perspective, we have seen the investigation of the noun class prefix and nominal agreement, consonants and clicks, acquisition of word order, relative clauses, morphophonology (for a review on studies of Bantu language acquisition, see Demuth [2003]; and for contemporary overview of studies on SA Bantu language, see Gxilishe, 2008; Pascoe & Smouse, 2012) and for pragmatic development, see Kunene-Nicolas (2015). It is apparent that linguists need to increase investigations on bilingualism and multilingualism which will feed into both research and language practice.

From the speech-language therapist and audiologist’s perspective, there is an acute need to provide assessment and intervention in the languages of their clients. There are many ways in which this can be accomplished, not least of which is the education and training of SA African language-speaking personnel as well as instruction in the African languages for those who are not mother tongue speakers. However, this is simply not enough. Deeper knowledge and understanding of the cognitive and linguistic processes inherent in these languages is essential if we are to provide the same level of service as we do in English and Afrikaans. This is particularly relevant to the literacy development of young children in the early school years and those who may be at risk for language impairment.

To reach this level of understanding it requires synergistic and nuanced research by multidisciplinary teams comprising inter alia linguists, psychologists and speech–language therapists. We believe that the articles contained in this volume illustrate this approach to research and provide excellent generalisable methods and results that can be applied to the study of other African languages and most importantly to the development of resources for assessment and intervention in these languages.

The article by Michelle Pascoe describes speech processing and production of 2-year-old children acquiring isiXhosa. Speech input processing, stored phonological knowledge and speech output are described based on data from auditory discrimination, naming and repetition tasks within a psycholinguistic framework. This article not only describes typical phonological development in isiXhosa but also provides a valuable theoretical framework and method within which future studies can be conducted.

The article by Nel and Southwood on the comprehension and production of quantifiers in isiXhosa-speaking Grade 1 learners focuses on a specific structure that children need to access and produce narratives and other classroom discourse. Little is known about the development, especially the production, of quantifiers, specifically in speakers of an African language. The study records the development of quantifiers in isiXhosa first language (L1) learners during Grade 1. By including two low socio-economic groups of L1 isiXhosa learners with either isiXhosa or English as language of learning and teaching (LOLT), the research takes the very important variable of context into account. Contextual influences of socio-economic status and language in education are critical in the South African context, and as expected this study showed that the isiXhosa LOLT group comprehended *no/none*, *any* and *all* fully either at the beginning or end of Grade 1, and produced all quantifiers at the beginning of the year. The English LOLT group had not acquired either the comprehension or the production of quantifiers by the end of Grade 1, although they did show some development. The English LOLT group performed worse than their peers who were taught in their L1. This article also illustrates the theoretical and methodological sophistication required to do research on African language acquisition.

Potgieter’s article on lexical and grammatical development in trilingual speakers of isiXhosa, English and Afrikaans provides much-needed normative data on linguistic development in multilingual children who constitute the largest part of the population who speak the African languages. The article also dispels negative lay opinion on the effect of early multilingualism on language development which ultimately affects home and school language policies. Focusing on isiXhosa, South African English and Afrikaans, the study involved a comparison of 11 four-year-olds developing trilinguals’ acquisition of vocabulary and passive constructions with that of 10 age-matched monolingual speakers of each language. The trilinguals proved to be monolingual-like in their lexical development in the language to which, on average, they had been exposed the most over time, that is, isiXhosa. No developmental delay was found in the trilinguals’ acquisition of the passive, regardless of the language of testing. As previously found for bilingual development, a reduced quantity of exposure does not hinder lexical development in the trilinguals’ input dominant language either. The overall lack of delay in their acquisition of the passive is interpreted as possible evidence of cross-linguistic bootstrapping, and support for early multilingual exposure. Once again, this article provides theoretical and methodological guidelines on the assessment of languages other than English and also provides evidence for value of raising children with more than one language. This has significant implications for policy and practice in education.

Wilsenach’s article on identifying phonological processing deficits in Northern Sotho–speaking children using non-word repetition is in line with current trends in language assessment practices internationally. Standardised language tests in the official languages of South Africa barely exist. Commercially available language tests are in English and have been standardised in other parts of the world. Such tests are often translated into African languages, a practice that speech-language therapists deem linguistically and culturally inappropriate. In response to the need for developing clinical language assessment instruments that could be used in South Africa, this paper reports on data collected with a Northern Sotho non-word repetition task. Non-word repetition measures various aspects of phonological processing, including phonological working memory, and is used widely by speech-language therapists, linguists and educational psychologists in the Western world. The design of a novel Northern Sotho non-word repetition task is described, and it is argued that the task could be used successfully in the South African context to discriminate between children with weak and strong Northern Sotho phonological processing skills, regardless of the LOLT. The non-word repetition task was piloted with 120 third graders and showed moderate to strong correlations with other measures of phonological working memory, such as digit span and English non-word repetition. Furthermore, the task was positively associated with both word and fluent reading in Northern Sotho, and it reliably predicted reading outcome in the tested population. Suggestions are made for improving the current version of the Northern Sotho non-word repetition task, whereafter it should be suitable to test learners from various age groups. Strong evidence is therefore provided for the use of this method as a potential assessment task in other African languages.

Kunene-Nicolas and Ahmed write on the lexical development of noun and predicate comprehension and production in isiZulu-speaking children aged between 25 and 36 months. It compares lexical comprehension and production in isiZulu, using a vocabulary assessment tool developed and validated in Italian: the Picture Naming Game developed by Bello *et al*. (2012). This study shows a detailed adaptation process of an assessment tool into the South African context and presents findings based on the direct assessment of the lexical inventory of 36 children. It confirms the universal theory that comprehension precedes language production. It highlights that children’s inventory is largely linked to their linguistic input in the home environment, and linguistic materials have to take this into account when children are introduced to a different language of learning in the formal school environment.

We are deeply grateful to the South African Speech Language and Hearing Association for the financial support in producing this special edition. We thank the Wits SPARC Fund for supporting the project.
